# Two Chairmen on the Crisis

**Published:** 1920-11-27

**Authors:** 


### TWO CHAIRMEN ON THE CRISIS.

Sir Ernest Hatch, Chairman of University College Hospital, and Lord Denbigh, Chairman of the Royal National Orthopædic Hospital, have both contributed to the discussion on the best way out of hospital financial and other difficulties. Their views are particularly useful as they represent the outlook of the moderate-sized hospital, which, in the nature of things, cannot be in such a desperate position as the London or any other of the giant institutions trying to weather the storm.

While Sir Ernest Hatch pays tribute to the splendid history of voluntary hospitals, he considers that their best friends should not close their eyes to the realities of the situation, the chief of which is that the existing hospitals are no longer adequate to the work they are called upon to do. If there were a fair prospect, he considers, of providing funds by voluntary means for maintaining our hospitals on a reasonable level of efficiency, there might be something to be said for carrying on on the old lines. But it is only too apparent that the high level of voluntaryism has been touched.

Thus far Sir Ernest Hatch announces himself as a member of the Knutsford school of thought, but he agrees that before any drastic change is made there should be a thorough and exhaustive inquiry.

But thorough and exhaustive inquiries do not pay current butchers' bills, and we are glad to see that Sir Ernest has a practical idea in suggesting that the Government or County Council should issue loans to hospitals at a low rate of interest to help them carry on until succour in some form is forthcoming.

Lord Denbigh also has a suggestion with which we must say at once that we are not much in love. It is that King Edward's Hospital Fund should be given funds by the Government to be allocated for general maintenance purposes to London hospitals. Provincial hospitals, it is suggested, are not quite in the same position as those in London, as by contributions from workpeople and in other ways it is thought they may carry on. In any case, Lord Denbigh intends his scheme to be considered only as a temporary measure of relief pending a final decision on the question as to whether the voluntary system can be preserved.

The carrying out of Lord Denbigh's proposal would add largely to the disciplinary powers of King Edward's Fund, which even now are not universally popular. Moreover, we doubt whether the Fund, not being a body elected by hospitals, would care to undertake such an onerous yet delicate task. Possibly, if repugnance to such a crude form of State aid were overcome, the Central Board that is so badly needed to represent the Metropolitan hospitals in their relationship with Government Departments and in other ways, might be of service in carrying out Lord Denbigh's plan. Then, quite properly, to a truly representative board, might very suitably be added delegates from the Sunday and Saturday Funds and King Edward's Hospital Fund.

It must be recorded that the principle of an inquiry was adopted by the meeting of members of both Houses of Parliament held on November 18, when it was resolved that "before any legislation is passed on the subject of State or rate aid for voluntary hospitals, an immediate inquiry into the whole subject should be held by an impartial Committee, and that such Committee should report at the earliest possible moment." Colonel Mildmay was requested to report the resolution to the Prime Minister and to the Minister of Health. The general view of the meeting was that, although the position is critical, an attempt of the Minister of Health to rush through a faulty solution was to be strongly challenged.

The London County Council and the Unionist Reconstruction Committee have both passed resolutions in favour of the proposed inquiry before legislation is considered.

